# Patterns and Predictors of Healthcare Use among Adolescent and Young Adult Cancer Survivors versus a Community Comparison Group

**DOI:** 10.3390/cancers13215270

**Published:** 2021-10-20

**Authors:** Ursula M. Sansom-Daly, Claire E. Wakefield, Christina Signorelli, Mark W. Donoghoe, Antoinette Anazodo, Susan M. Sawyer, Michael Osborn, Rosalie Viney, Nicholas Daniell, Kate Faasse, Richard J. Cohn

**Affiliations:** 1 School of Women’s and Children’s Health, UNSW Medicine and Health, UNSW Sydney, Kensington, NSW 2052, Australia; c.wakefield@unsw.edu.au (C.E.W.); c.signorelli@unsw.edu.au (C.S.); m.donoghoe@unsw.edu.au (M.W.D.); antoinette.anazodo@unsw.edu.au (A.A.); r.cohn@unsw.edu.au (R.J.C.); 2 Kids Cancer Centre, Behavioural Sciences Unit, Sydney Children’s Hospital, Randwick, NSW 2031, Australia; nicholas.ca.daniell@gmail.com; 3 Sydney Youth Cancer Service, Prince of Wales Hospital, Randwick, NSW 2031, Australia; 4 Stats Central, Mark Wainwright Analytical Centre, UNSW Sydney, Kensington, NSW 2052, Australia; 5 Department of Paediatrics, The University of Melbourne, Melbourne, VIC 3052, Australia; susan.sawyer@rch.org.au; 6 Royal Children’s Hospital Centre for Adolescent Health, Melbourne, VIC 3052, Australia; 7 Murdoch Children’s Research Institute, Melbourne, VIC 3052, Australia; 8 Youth Cancer Service SA/NT, Royal Adelaide Hospital, Adelaide, SA 5000, Australia; michael.osborn@sa.gov.au; 9 Centre for Health Economics and Research and Evaluation (CHERE), University of Technology Sydney, Sydney, NSW 2000, Australia; rosalie.viney@chere.uts.edu.au; 10 School of Psychology, UNSW Sydney, Kensington, NSW 2052, Australia; k.faasse@unsw.edu.au

**Keywords:** cancer survivorship, cancer survivors, survivorship, healthcare utilization, adolescent, young adult, neoplasms/psychology, mental health services, psychosocial factors, psychotropic medication

## Abstract

**Simple Summary:**

Adolescent and young adult cancer survivors face several significant physical/mental health late effects following cancer treatment. These effects may be minimized through healthcare use tailored to young survivors’ needs. Using a cross-sectional study design, we examined the healthcare use of 93 adolescent/young adult cancer survivors (aged 15–39 years), relative to a comparison group of adolescents and young adults recruited from the local community (*n* = 183). Our cancer survivor group reported greater use of medical and mental health services, and medications during the past six months relative to the comparison group. Our cancer survivor group also reported less psychological distress, and similar work/study participation relative to the comparison group. Survivors who were female, diagnosed with brain/solid tumors and who had finished treatment more recently reported greater healthcare use. Future research is needed to determine whether the healthcare accessed by adolescent and young adult cancer survivors is appropriate and meets their needs.

**Abstract:**

Healthcare use (HCU) during survivorship can mitigate adolescent and young adult (AYA) cancer survivors’ (aged 15–39 years) risk of medical and psychosocial late effects, but this is understudied. We surveyed 93 Australian AYA post-treatment cancer survivors (*M_age_* = 22.0 years, SD = 3.5; 55.9% female) and a comparison sample of 183 non-matched AYAs (*M_age_* = 19.7, SD = 3.2; 70.5% female) on their HCU, medication use, depression/anxiety, and general functioning. Relative to our comparison AYAs, a higher proportion of our survivor group reported medical HCU (community-delivered: 65.6% versus 47.0%, *p* = 0.003; hospital-delivered: 31.2% versus 20.3%, *p* = 0.044) and mental HCU (53.8% vs. 23.5%; *p* < 0.0001) in the past six months. A higher proportion of our survivors reported taking medications within the past six months than our comparison AYAs (61.3% vs. 42.1%, *p* = 0.003) and taking more types (*p* < 0.001). Vitamin/supplement use was most common followed by psychotropic medications. Our survivor group reported lower depression (*p* = 0.001) and anxiety symptoms (*p* = 0.003), but similar work/study participation (*p* = 0.767) to our comparison AYAs. Across groups, psychological distress was associated with higher mental HCU (*p* = 0.001). Among survivors, those who were female, diagnosed with brain/solid tumors and who had finished treatment more recently reported greater HCU. Future research should establish whether this level of HCU meets AYAs’ survivorship needs.

## 1. Introduction

The diagnosis of cancer in an adolescent or young adult (AYA, aged 15–39 years consistent with the broadest global definition) risks fundamentally disrupting their developmental trajectory towards adulthood [1,2,3], compounded by adolescence and young adulthood being the life stage across which mental health disorders are most likely to emerge [4]. Worldwide, over one million AYAs grapple with this new reality each year [5]. Fortunately, international estimates suggest that up to 88% can expect to survive their disease and move towards longer-term survivorship and life as an adult [5]. This means that ensuring the physical and mental health and well-being of young cancer survivors is an important priority.

AYAs face a range of complex physical and psychosocial late effects that can last decades following completion of cancer treatment. This includes physical late effects (e.g., endocrine and cardiopulmonary damage) [6], the potential for recurrent or second primary cancers [7,8], complex mental health effects including depression, anxiety, post-traumatic stress [3,9,10], and higher rates of fear of cancer recurrence than older patients [11,12]. AYAs’ young age at diagnosis means that the impact of their physical and mental health late effects on their general functioning in survivorship can be profound. Economic data from Australia show that the loss of productivity and future potential among AYA cancer patients diagnosed every year costs the economy an estimated AU $455 million in lifetime costs, far eclipsing the cost of the cancer treatment itself [13].

In addition to AYA survivors’ profile of psychological late effects, their lack of high-level health literacy [14] and documented desire to avoid mental health-related stigma [15] may impact how they access and interact with healthcare services [16]. International consensus supports the idea that AYAs living with and beyond cancer may benefit from tailored, age-appropriate healthcare services to address their unique needs [17,18,19,20,21,22,23]. Indeed, AYAs report desiring healthcare services that are tailored to their developmental needs [24,25]. Better understanding by AYAs of their unique risk profile for treatment-related late effects and cancer recurrence and timely access to specialist AYA services may also minimize the burden of future physical and psychological late effects [26,27,28]. Engaging AYAs in age-appropriate, tailored, multidisciplinary survivorship care is therefore key to ensuring their physical and mental health as they mature.

In Australia, cancer survivors in the AYA age range are typically managed either by ‘long-term’ follow-up clinics based within pediatric (children’s) hospitals for long-term survivors (beyond five years post-diagnosis) [27,29], or through the survivorship clinics of the national network of Youth Cancer Services [30]. The Youth Cancer Services provide survivorship care tailored to AYAs diagnosed between the ages of 15 and 25 years, across pediatric hospitals (for AYAs diagnosed between 15 and 17 years old) and adult hospitals (for AYAs diagnosed at 18 years and older) [30]. The positive, age-appropriate communication and support experiences that these services can facilitate appear to lead to improved quality of life [21,24,31] and may foster greater engagement into long-term cancer survivorship than traditional, medically-driven models of care by accounting for the particular psychosocial needs of AYAs [27,29,32,33,34,35]. 

Existing research has focused on quantifying the impact of youth-friendly, age-appropriate healthcare for AYAs during active cancer treatment. Less research has reported on the healthcare services accessed by AYA cancer survivors after cancer treatment completion and further into survivorship [26]. More data are currently available about long-term survivors of childhood cancer, up to three-quarters of whom appear not to use recommended, cancer-related follow-up services [26,29]. Review data recently identified that while 65% of long-term childhood cancer survivors engaged in some form of healthcare use in relation to their survivorship (HCU), this ranged considerably, from 36 to 89% [26]. Survivors who were female, had received radiation therapy, were further from diagnosis, and who reported having a higher income, greater educational attainment, and higher self-reported health-related quality of life reported increased HCU [26]. 

Individual factors accounting for which AYAs engage with what types of health services in survivorship also remain largely unexplored. One early study reported that AYA cancer survivors who identified more closely with the term ‘cancer survivor’ were more likely to access professional mental health services [36]. This suggests that elements related to how AYAs have psychologically responded to their cancer experience may be reflected in their patterns of HCU. There are documented impacts of the diagnosis, treatment, and symptoms of cancer on AYAs’ later education and occupational productivity and attainment [2,37]. However, little research has examined patterns of HCU, mental health, and survivor functioning in these domains. Consequently, it remains unclear whether and how AYAs’ well-being and general functioning in survivorship is associated with the extent to which they access specialized medical and supportive services following their cancer treatment. 

This study aimed to examine how a cohort of Australian AYA cancer survivors accessed healthcare to inform how services might best support AYAs to engage with developmentally-appropriate healthcare in survivorship. We anticipate that these data can inform how both hospital-delivered, AYA-specific health services and community-delivered (generalist) health services might be better tailored to target the most ‘at risk’ survivors, and most effectively mitigate the potential for adverse medical and psychological late effects. Given the predominant literature is from North America with a particular set of barriers to HCU [26], it is also important to report on how AYA survivors are faring and functioning in countries with different healthcare systems.

With reference to a locally-recruited, community-based comparison group, we aimed to describe AYA cancer survivors’ (1) overall HCU, (2) medical HCU (e.g., oncologist, general practitioner (GP), nurse consultations), (3) mental HCU (e.g., psychologist, social worker, psychiatrist consultations), and (4) medication use. Across each outcome, we examined whether individual differences according to sociodemographic and cancer-related factors were associated with HCU. Finally, we examined whether (5) the extent to which AYA cancer survivors engage in HCU in survivorship was associated with their subsequent general functioning, quality of life, or mental health.

## 2. Materials and Methods

This cross-sectional study was approved by the South Eastern Sydney Local Health District (SESLHD) Human Research Ethics Committee (Reference: HREC/12/POWH/136) as well as the Human Research Ethics Advisory Panel C (Behavioural Sciences), UNSW Sydney (File 2892).

### 2.1. Eligibility Criteria

In order to capture diverse cancer survivorship experiences and broad representation of the spectrum of HCU in AYAs surviving cancer, we recruited AYAs currently aged 15–39 years old, consistent with the broadest international definition [38], all of whom had completed cancer treatment at least one month prior, consistent with a post-treatment definition of ‘survivorship’. AYAs could have been diagnosed prior to the age of 15 but were now in the AYA age group. We included AYAs recruited from two survivorship clinics at a major metropolitan pediatric/AYA cancer service, including a post-treatment follow-up clinic and a long-term survivorship cohort (which provides survivorship care for survivors beyond five years post-diagnosis). We also recruited a convenience sample of AYAs aged 15–39 years with no history of cancer from the local community to function as a comparison group (see below). AYAs who did not have sufficient English language skills to complete the questionnaire were ineligible.

### 2.2. Recruitment

We invited AYA cancer survivors through the Sydney Children’s Hospital, Australia long-term follow-up clinic lists, as well as the Sydney Youth Cancer Service patient database which also included patients from Prince of Wales Hospital Australia. We mailed cancer survivor participants a study package including a personalized invitation letter from an oncologist at that site, information sheet, consent form and questionnaire. Participants had the option to complete the questionnaire either on paper or using an online link. 

We recruited our community-based comparison group of AYAs with no history of cancer through several sources, including undergraduate psychology students who participated in return for partial course credit, as well as through poster advertisements displayed on a local university campus (UNSW Sydney), a local gymnasium frequently attended by high-school students, and through the newsletters of several local public and private high schools. This convenience sample was drawn from the same geographical area as the cancer survivor cohort but was not matched for any other characteristics. This pragmatic recruitment strategy was designed to obtain a comparison AYA sample that was geographically well-matched, but did not strictly control for matching on other variables such as age (with our comparison AYAs recruited from local educational institutions more likely to be younger in age). We sent interested community participants who responded to the advertisement a study package containing an invitation letter, participant information sheet and consent form, and study questionnaire. 

### 2.3. Measures

A multidisciplinary team of psychologists, oncologists, and health economists purposely designed the questionnaire which assessed AYAs’ health service and medication use, as well as general functioning (including participation in work/study and other productive activities), quality of life, and mental health. Table 1 summarizes the battery of measures used.

### 2.4. Data Analysis

We compared participants’ demographic characteristics across groups (cancer survivors vs. comparisons) using independent t-tests and chi-squared tests for continuous variables and categorical variables, respectively. We performed Pearson chi-squared tests on the proportion of AYAs accessing each type of healthcare service. 

We then examined two aspects of survivors’ HCU: their overall access (whether or not they had ever used a particular service in the 6 month period; a binary yes/no outcome), and their intensity of use (how many service types or occasions of service within a particular healthcare service category they had accessed in that period; a continuous outcome). For simple descriptive summaries of HCU according to survivors’ time since treatment-completion, we categorized survivors according to whether they were in their first, second–fourth, or fifth year and beyond treatment completion (i.e., long-term cancer survivorship) as each of these stages has distinct healthcare-related needs and recommendations for surveillance and follow-up care. In subsequent regression analyses, we used time since diagnosis as a continuous predictor variable in order to retain information that would be lost through categorizing [44]. We categorized survivors’ age at diagnosis according to whether they were diagnosed at a ‘pediatric age’ (under 15 years) or as an ‘AYA’ (15 years and older, the age where AYAs with cancer are typically managed through AYA-specific Youth Cancer Services in Australia). 

To investigate overall access, we used multivariable binary logistic regression analyses to investigate factors associated with AYA cancer survivors’ HCU. We included several sociodemographic and cancer-related variables associated with AYA survivorship needs and outcomes as covariates across all regression models. Several additional binary predictor variables were created from participant demographics to facilitate logistic regressions predicting AYAs’ likelihood of reporting HCU across the five categories above (see Table 1). Multicollinearity tests were undertaken at the outset to avoid problematic collinearity between these independent predictors. The variance inflation factor (VIF) was examined for each independent variable with a VIF ≤ 3 considered acceptable. Problematic multicollinearity emerged between education status, and age at diagnosis and time since treatment; we therefore removed education as a predictor because its association with HCU was not of primary interest compared to obtaining more precise estimates of the other variable coefficients in the model. Consequently, our final analyses included these covariates: sex, employment status, speaking a language other than English at home, age at cancer diagnosis, diagnosis category (blood cancer vs. not), and time since treatment completion. 

We used these predictors to examine HCU according to the following categories within logistic regression models (i) overall HCU, (ii) medical HCU (including GP, oncologist, nurse, fertility specialist and hospital Emergency Department visits, as well as any hospital admissions), (iii) hospital-delivered HCU only (hospital Emergency Department visits, as well as any hospital admissions only), (iv) mental HCU (including psychologist, psychiatrist, social worker, counsellor, and community-delivered support), and (v) medication use. Medication data were categorized according to any use, and number of medications taken, with medication and supplement use categorized according to the Monthly Index for Medical Specialties (MIMS) Online Database. 

We used these same predictor variables within subsequent Poisson regression models to examine the extent to which these sociodemographic and cancer-related variables accounted for survivors’ total number of self-reported healthcare service use within each of these same HCU categories. 

Finally, in order to examine whether AYAs’ extent of engagement with HCU during the past six months was associated with their current health-related quality of life or general functioning, multivariable linear regression analyses were carried out that accounted for sociodemographic and cancer-related factors. Given the relative lack of data in this area, we took a hypothesis-generating approach, and did not control for multiple comparisons.

## 3. Results

### 3.1. Participant Characteristics

We recruited 276 AYAs (93 cancer survivors, 183 comparison AYAs) prior to the onset of the global Coronavirus (COVID-19) pandemic. We were not able to calculate a response rate in either sample due to our use of open advertisements in recruiting our community-based comparison group, and ethical approval restrictions from having recruited cancer survivors across multiple hospitals [45].

Table 2 depicts participant characteristics by group. Cancer survivors were 22.0 years old on average (*SD* = 3.5), with a median age at diagnosis of 16 years (range: 0–27 years; Table 2). The cancer diagnoses represented were broadly representative of recent national registry-based data [46], with a slight over-representation of blood cancers relative to typical AYA patient samples, which will to some extent reflect our recruitment from a pool of AYA-aged cancer survivors diagnosed before 15 years old. Approximately two-thirds of the sample were within five years post-diagnosis. Due to the pragmatic, convenience-sampling approach we took in recruiting our comparison group, they were slightly younger (*M_age_* = 19.7, SD = 3.2 vs. *M_age_* = 21.9 years, SD = 3.5; *p* < 0.001), and had a higher proportion of female respondents (70.5% vs. 55.9%, *p* = 0.036). Our comparison AYAs also had lower proportions who had completed post-high-school education (12.6% vs. 51.6%; *p* < 0.001), but more who were currently studying or employed (94.5% vs. 87.1%; *p* = 0.031) relative to the AYA cancer survivors. 

### 3.2. Healthcare Use (HCU) Outcomes

Table 3 depicts rates of overall HCU, and for medical, mental health, and medication categories for the AYA cancer survivor group relative to the community-based comparison group. Table 4 depicts patterns of HCU across these same categories for cancer survivors alone, according to their age at diagnosis and the length of time since completing active cancer treatment.

#### 3.2.1. Overall HCU

##### Patterns of Use (Rates)

Our AYA cancer survivor group reported similar rates of overall HCU as the comparison group over the past six months (65.6% vs. 60.1%; *χ^2^* = 0.786, *p* = 0.375). Within the cancer survivor group, those diagnosed as an AYA reported higher rates of overall HCU relative to survivors diagnosed at a pediatric age (84.5% vs. 34.3%; *χ^2^* = 24.370, *p* < 0.001; Table 4). Examined according to time since cancer treatment completion, a clear and significant pattern emerged; AYAs in their first year post-treatment reported the highest overall HCU (90.5%), which declined steadily for survivors in their second (77.8%), third or fourth (61.5%) or fifth or more years post-treatment (i.e., long-term survivors; 27.6%; *χ^2^* = 30.770, *p* < 0.001; Table 4).

##### Factors Associated with HCU among Cancer Survivors 

Access (any use): Table 5 depicts univariable and multivariable regressions accounting for all HCU outcomes within the cancer survivor group. Multivariable analyses indicated that survivors who were younger at diagnosis (*OR* = 0.72; *CI* = 0.55–0.96; *p* = 0.023), were diagnosed with a non-blood cancer (*OR* = 9.79; *CI* = 1.48–64.87; *p* = 0.018) and had completed treatment more recently (*OR* = 0.95; *CI* = 0.92–0.98; *p* < 0.001) were more likely to report accessing any type of HCU. These findings indicated that the likelihood of survivors reporting recent HCU decreased as both survivors’ age at diagnosis and time since treatment completion increased (*p* < 0.001) and suggested that survivors of non-blood cancers (mostly solid and brain tumors) were more likely than survivors of blood cancers to report HCU in the past six months. 

#### 3.2.2. Medical HCU

##### Patterns of Use (Rates)

Our cancer survivor group reported higher rates of medical HCU than our comparison group in the last six months (66% versus 47%; *p* = 0.003). The two groups reported engaging with GP services at similar rates, with approximately one-third of each group reporting a GP visit during the past six months (*p* = 0.904; Table 3). Survivors reported higher rates of hospital-delivered HCU (31%) relative to comparisons (20%; *χ^2^* = 4.074; *p* = 0.044), including emergency department presentations (25% versus 13%; *χ^2^* = 5.889; *p* = 0.015; Table 3). 

AYA cancer survivors who had been diagnosed at an AYA age reported higher rates of medical HCU overall compared with survivors diagnosed at a pediatric age (75.9% vs. 48.6%; *χ^2^* = 24.370, *p* < 0.001; Table 4). This same pattern emerged for AYAs’ recent consultations with nurses, fertility specialists, and other health professionals, though not for GPs (see Table 4). Survivors diagnosed as AYAs also reported more recent emergency department admissions relative to survivors diagnosed at a younger age (32.8% vs. 11.4%; *χ^2^* = 5.335, *p* = 0.021; Table 4).

Examined according to time since treatment completion, AYAs who had completed treatment most recently were more likely to report having accessed any medical HCU overall (*χ^2^* = 9.362, *p* = 0.025; Table 4) as well as having seen their oncologist in the past six months, relative survivors diagnosed further ago (*χ^2^* = 9.302, *p* = 0.026; Table 4). Almost half of survivors who were within their first year post-treatment reported having seen their oncologist in the past six months (45.2%), a rate that steadily declined with each year post-treatment, reaching 13.8% for survivors beyond five years post-treatment (Table 4). AYAs in their first-year post-treatment were most likely to report having seen a nurse recently (40.5%), a rate that sharply declined for all subsequent years post-treatment (to 3.4% at 5 years post-treatment; *χ^2^* = 19.749; *p* < 0.001). A similar proportion of survivors reported having seen a GP recently, regardless of time post-treatment (ranging from 31.0 to 44.4% across all years post-treatment), which did not significantly differ with greater time since treatment (*χ^2^* = 0.0635, *p* = 0.888).

##### Factors Associated with HCU among Cancer Survivors

Access (any use):Within adjusted multivariable analyses, female survivors were more likely than their male counterparts to report accessing medical HCU during the past six months (OR = 4.38; CI = 1.42–13.58; *p* = 0.010; Table 6). No other sociodemographic or cancer-related factors were associated with access to medical HCU. 

Intensity of usage (number of types): Time since treatment was the only factor associated with greater HCU; survivors who had completed treatment more recently reported greater HCU overall *(χ^2^* = 9.707, *p* = 0.002; Table 6), as well as specifically within medical HCU (*χ^2^* = 7.189, *p* = 0.007) and hospital-delivered HCU (*χ^2^* = 7.942, *p* = 0.005). 

#### 3.2.3. Mental Health, and Mental HCU

##### Depression, Anxiety, and Perceived Health

A minority of all participants reported moderate or greater levels of depression and/or anxiety during the past week (*n* = 44/276, 15.9%). Our cancer survivor group had lower average depression and anxiety scores than the comparison group (*p*-values ≤ 0.003; Table 7). Furthermore, survivors were less likely than those in the comparison group to report current anxiety symptoms in the moderate-to-severe range (*OR* = 0.31, *95%CI* = 0.10–0.93, *p* = 0.037). The groups did not differ on their self-assessed current health-related quality of life, indicating similar perceptions of satisfaction with their overall health (*χ*^2^ = 1.30, *p* = 0.255; Table 7).

##### Missed Study/Work and Productivity

Approximately half of both groups reported having taken at least some time off from paid work or study in the past month, and the estimated days of missed study/work (time off) was comparable across groups (Table 7). The nominated reasons for these absences differed between groups. The probability of those in the survivor group attributing time taken off from work or study due to ‘self-consciousness and concerns about physical scars’ was higher than in the comparison group (*p* < 0.001). By contrast, the probability of those in the comparison group attributing their time taken off from work/study as being due to ‘tiredness or low energy’ (*p* = 0.023) and/or feeling ‘unable to keep up with the workload’ (*p* = 0.045) was higher than in the survivor group. 

Overall, the survivor group also reported engaging in productive activities across more days during the past month than the comparison group (M = 15, SD = 6.3 vs. M = 12.4, SD = 5.6 days; *p* < 0.001; Table 7). The focus of these activities appeared to differ between groups. Survivors reported relatively greater engagement in personal hobbies (*p* = 0.002), and exercise/sports (*p* = 0.028) than the comparison group. By contrast, the comparison group reported more time spent engaging in study (*p* < 0.001), and group social activities, including activities such as team sports, university/college-run clubs and societies, and youth groups (including religious groups) than the cancer survivor group (*p* = 0.007).

##### Patterns of Use (Rates)

A higher proportion of our AYA cancer survivors reported using mental health services than our comparison group (52.7% compared with 23.5%; *p* < 0.001; Table 3). Within the cancer survivor group, survivors reported the highest rates of overall mental HCU in their first year post-treatment (73.8%) which declined over all subsequent years post-treatment (to 27.6% beyond 5 years post-diagnosis; *χ^2^* = 18.058, *p* < 0.001; see Table 4). A similar pattern emerged for our survivors’ use over time for psychologists (*χ^2^*=10.346, *p* = 0.016), social workers (*χ^2^* = 26.400, *p* < 0.001), counsellors (*χ^2^* = 13.597, *p* = 0.004), and community-delivered support organizations (*χ^2^* = 16.946, *p* < 0.001; see Table 4). A small proportion of survivors reported engaging with psychiatrists, and this did not differ according to time since completion of treatment (*χ^2^* = 2.119, *p* = 0.548). 

Supplementary Table S1 details overall mental HCU, and mental HCU according to service/discipline type, according to participant group and distress level. Across cancer survivor and comparison groups, AYAs whose DASS-21 scores were in the higher range (moderate to extremely severe symptom severity) were more likely to report accessing any mental HCU during the past six months relative to less distressed AYAs (*χ^2^* = 10.599, *p* = 0.001). Yet, less than half of these distressed AYAs had not accessed any mental healthcare during the past six months (45.5%; Supplementary Table S1). While more distressed AYAs appeared more likely to report mental HCU, the specific type of mental HCU they accessed did not appear to differ as a function of their distress levels; across both cancer survivor and comparison groups, AYAs with greater and lesser distress reported having seen psychologists (χ^2^ = 2.403, *p* = 0.169), psychiatrists (χ^2^ = 0.469, *p* = 0.557), social workers (*χ*^2^ = 0.022, *p* = 0.883), counselors (*χ*^2^ = 0.795, *p* = 0.372) and GPs (*χ*^2^ = 1.435, *p* = 0.231) at similar rates during the past six months.

##### Factors Associated with Mental HCU among Cancer Survivors

Access (any use): Within adjusted multivariable analyses, only cancer diagnosis appeared to account for whether or not survivors reported mental HCU (Table 5). Relative to survivors of blood cancers, survivors of non-blood cancers were more likely to report mental HCU in the last six months (*p* = 0.030). 

Intensity of usage (number of types): The Poisson regression analysis revealed that only time since treatment completion significantly accounted for intensity of mental HCU during the past six months. Survivors who had completed treatment more recently reported having accessed more types of mental HCU (*χ*^2^ = 7.407, *p* = 0.006; Table 6). 

#### 3.2.4. Medication Use

##### Patterns of Use (Rates)

Those in our survivor group were more likely to self-report using at least one medication during the past six months relative to our comparison AYAs (*p* = 0.003) and reported taking a higher number of medications on average over this period (*M* = 1.8 versus 0.7, *p* < 0.001, Table 3). A range of medications were described (see Table 8). Vitamins and supplements were the most commonly reported category identified across both groups (reported by 18.3% of survivors and 16.9% of comparison AYAs). Survivors reported higher rates of using psychotropic anti-depressant/anxiety medications (11.8% vs. 5.5%) and pain medications (7.5% vs. 1.1%) relative to the comparison group, including opioid or opioid-like analgesics such as endone, oxycodone, and tramadol hydrochloride. A subset of both groups, all of whom were female, reported using medication-based contraceptives (19.2% of female survivors, 16.9% of female comparison AYAs). The small cell numbers in these data prohibited a more granular statistical analysis of specific medications.

When psychotropic medications were examined, across both groups, AYAs who reported having ever seen a psychologist, psychiatrist, or counselor at any time in the past (prior to study intake) were more likely to report having used psychotropic medications during the past six months (*F* = 12.991, *p* < 0.001). We also examined the relationship between AYAs’ current distress levels and their use of psychotropic medications. AYAs who reported *DASS-21* depression and/or anxiety scores in a moderate or higher range were more likely to report also using psychotropic medications during the past six months (*χ^2^* = 12.287, *p* < 0.0001) than AYAs reporting low anxiety and/or depression scores.

We also examined the extent to which patterns of psychotropic medication use occurred in conjunction with other forms of mental HCU (Supplementary Table S2). Across the whole sample, AYAs who reported taking some form of psychotropic medication during the past six months were also more likely to report having seen either a psychiatrist or GP during this time (χ^2^ = 22.758, *p* < 0.001). Three AYA survivors (3/21; 14.3%) using psychotropic medication did not report either having seen a psychiatrist or a GP during this same period in the last six months. 

Among the survivor group only, neither AYAs’ age at diagnosis (t = −0.303, *p* = 0.763) nor their length of time since treatment completion (χ^2^ = 1.174, *p* = 0.759) appeared to impact their likelihood of reporting recent medication use (Table 4).

##### Predictors of Use among Cancer Survivors

Access (any use): Across univariable and multivariable analyses, no variables appeared to be associated with survivors’ likelihood of reporting recent medication use (when medical contraceptive use was excluded; Table 6). 

Intensity of usage (number of types): No sociodemographic or cancer/treatment-related factors were significantly associated with the number of medications survivors reported taking. AYAs’ length of time into survivorship did not significantly impact the rates at which they reported using any medications, with early- and longer-term survivors reporting this at similar rates (*χ^2^* = 1.174, *p* = 0.759; Table 4).

#### 3.2.5. Relationship between Greater HCU, Health-Related Quality of Life and General Functioning among AYA Cancer Survivors

##### Engagement with HCU and Perceived Health-Related Quality of Life Status

Taken together, AYA survivors’ recent degree of engagement in overall HCU and their cancer diagnosis type were both independently associated with their perceived health-related quality of life. Poorer perceived health-related quality of life was associated with fewer reported types of recent HCU (t = −2.558, p = 0.012) and a blood cancer diagnosis (t = −2.078, p = 0.041; Supplementary Table S3).

##### Relationship between HCU and Missed Study/Work and Productivity

Adjusted multivariable analyses revealed that the extent to which survivors had accessed different types of HCU recently, together with participant sex, was associated with engagement with work/study (Supplementary Table S3): more days engaged in work/study were observed among female AYA survivors (*t* = 2.252, *p* = 0.027) and those who reported accessing fewer types of HCU recently (*t* = −2.256, *p* = 0.027).

## 4. Discussion

At the completion of cancer treatment, it is important that AYA cancer survivors have the best possible opportunity to rejoin their peers on the developmental trajectory towards independent, well-functioning adults. Understanding how AYAs continue to use the healthcare system to assist them on this path plays an important role in optimizing survivors’ medical and psychological well-being in the years after cancer has been successfully treated. Relative to a community-based comparison group, our cohort of AYA survivors reported higher total HCU, including greater medical and mental HCU, which was especially prominent in the first few years after completing active cancer treatment. They accessed specialist and hospital-delivered services more often than GPs. These young survivors were more likely than those in our comparison group to have recently engaged with mental health services, and to be taking more medication. Our survivors also appeared to be functioning well. On average, they were less distressed than the comparison subjects, with similar rates of participation in work or study and higher engagement with hobbies, sports and group social activities. Although the comparison group was drawn from a non-matched convenience sample, these data may indicate that our survivor sample had recovered somewhat from the cancer-related educational and vocational goal disruption that has been linked to poorer mental health and quality of life long-term [2,47,48].

Across analyses, several factors repeatedly emerged as being associated with greater HCU among survivors: more recent treatment completion, female sex, and being diagnosed with a non-blood cancer (i.e., brain or solid tumor). Our finding that HCU decreased as the time since treatment increased echoes previous research [26,49,50,51,52]. Given that survivors are at increased risk of late effects the further from treatment they are [50]. the reduced HCU during this period may represent a significant gap in care. Further, unlike in other countries such as the United States (US), in Australia hospital-delivered services are largely free of financial cost for survivors; this means that in contrast to US data [33], cost may not have been a considerable barrier for the hospital-delivered, medical HCU at least. Brain and solid tumor survivors, and female survivors, were groups who continued to access medical and mental HCU to a greater extent into survivorship. This may indicate that healthcare services may need to devote particular efforts to continue to effectively engage male survivors, as well as survivors of blood cancers, over time. 

Echoing other recent research [26], our study is unable to determine the extent to which the medical or mental HCU accessed in survivorship was appropriate. The finding that females had accessed more care raises questions around whether those accessing services are those who need it most. Previous studies have reported that male AYA cancer survivors are more likely to experience higher levels of unmet information needs [53,54], and yet are significantly less likely to access medical care into long-term survivorship [49,52]. Pleasingly, we found that AYAs who were currently more distressed had engaged with mental HCU to a greater degree. We also found that among our survivors, greater overall HCU was associated with better self-reported health-related quality of life. These patterns mirror several other studies [26,55,56]. While our cross-sectional data cannot determine causality, this pattern may indicate that the healthcare being accessed was to some extent achieving its goals: that is, that AYAs’ recent HCU was facilitating improved perceived health overall, with mental healthcare targeted towards, and accessed by, individuals currently distressed and in need of healthcare. 

The higher utilization of mental healthcare services by our AYA survivor cohort relative to our comparison group may have contributed to their lower self-reported distress. In Australia, data highlight a considerable gap in mental healthcare for AYAs with mental health problems in the general community: while approximately 25% access some form of treatment, fewer than 2% receive specific help from mental health specialists [57]. Encouragingly, our data appeared to show less evidence of a mental health treatment gap among our survivor group relative to comparison AYAs: survivors’ mental HCU appeared to be relatively well matched with their distress levels, as a greater proportion of highly-distressed AYA survivors reported mental HCU relative to less distressed AYAs. By contrast, over half of the high-distressed comparison AYAs reported no mental HCU in the past six months. This was the case across several disciplines of mental health professionals, including social work and psychology. It may be that our survivor group benefited from greater access to hospital-delivered, cost-free psychology and social work services into survivorship [30,58].

The finding that our survivor group showed particularly high rates of mental HCU in the early few years post-treatment, which decreased with time, may also indicate that hospital-delivered services were serving as critical gateways to, and/or providers of, comprehensive screening and referral to age-appropriate mental health interventions. Our survivor group also reported greater use of support organizations in the community, some of which offer counselling services and may also have addressed these mental health needs. These findings stand in contrast to reports of a lack of access to age-appropriate, mental health services available to AYAs without cancer in the state of New South Wales [59].

In this study, psychotropic medication usage seemed to be relatively well aligned with anxiety and depression scores in our survivor sample, in contrast to a substantial gap between the two in the comparison sample. Despite our survivors’ overall positive general functioning outcomes and lower distress, almost 12% of them reported currently taking anti-depressant and/or anti-anxiety medications, double the rate in our comparison group (5.5%). This rate was somewhat lower than recent US data showing that 22% of long-term childhood cancer survivors reported currently using psychotropic medications however [60]. Prior research has highlighted that AYA cancer survivors may take antidepressant medications at rates 20–38% higher than their peers [61,62]. Considering our survivor cohort’s relatively lower distress, it may be that this cohort of survivors was somewhat ‘over-prescribed’, or that prescription use has lingered without being monitored or adjusted. It may also reflect gaps in accessing pharmacological treatment in the community comparison group however. Pharmacological treatments for mental health disorders are indicated at the higher end of distress, after other treatments have been pursued (e.g., psychological ‘talking therapies’ such as cognitive-behavioral therapy) [63]. It is possible that for these survivors, the pharmacological treatment had effectively treated their symptoms, but had not yet been reduced or weaned. That a minority of our survivor cohort were taking psychotropic medications without concurrent engagement in psychological talking therapies is contrary to recommendations within guideline-driven care, and highlights opportunities for improving clinical care. 

Finally, our data also highlighted that vitamins/supplements were the most commonly-reported ‘medication’ type across both survivor and comparison-group AYAs. This is consistent with recent research which found 42% of cancer survivors ranging from childhood to young adulthood reported using non-pharmacological or natural therapies, such as herb/supplement mixtures [64]. While some of this use may be appropriate and indicated (e.g., zinc in the case of vitamin deficiency), some may not, and may be not evidence-based. Our data cannot reveal the motivations behind this use. While it may reflect a more holistic orientation to ‘wellness’, greater use of complementary and alternative medicines can also reflect ongoing health-related concerns, such as higher fear of cancer recurrence [65]. Given their relative cost, understanding the extent of, and motivations for, vitamin/supplement use in young cancer survivors is an important point for future research.

### 4.1. Strengths and Limitations

This paper is one of the first reports to provide insights into the long-term use of healthcare and general functioning of Australian AYA cancer survivors, relative to a comparison group from the general community who have not had cancer. Our questionnaire probed survivors’ patterns of self-reported healthcare use in considerable detail, which adds to existing knowledge available through registry-based studies [53,54]. Several study limitations warrant consideration. Our sample was modest in size relative to international, registry-based cohort samples, and our convenience-based comparison group was not matched to our survivor group other than their recruitment from the same geographical catchment area. In particular, the higher proportion of females in our comparison group may have influenced the rates of HCU seen in our cohort of AYAs without cancer. Our comparison group was also more diverse than our cancer survivor cohort in terms of country of birth and language spoken at home. While this may reflect the high degree of diversity found in the Australian university sector (from which many of our comparisons were recruited) [66], it may also point to the relative lack of representativeness of the cancer survivors, likely at least in part due to our eligibility criteria requiring English fluency, as contemporaneous data indicated that approximately 58% of 12–24 year-old hospital patients from within the local health district our survivors were recruited from spoke English as a first language (81,659/140,911; Personal Communication, May 2018) [67]. Our comparison group may not have been completely representative of the Australian population of AYAs without cancer. Given that we recruited through a local university, our comparison group may have been more highly educated and of a higher socioeconomic status than the general AYA population. Observed differences in the two groups may therefore have been partly a result of our recruitment strategy.

Our questionnaire method relied upon retrospective self-report which is subject to bias and inaccuracies. Though comprehensive, our questionnaire did not ask about several specialist health professions (e.g., dental care) that may also be important for long-term medical care in survivorship. While we recruited our survivor group from a metropolitan hospital with a co-located AYA-specific Youth Cancer Service, we did not ask survivors to specify where they accessed services from (e.g., a hospital-delivered psychologist versus private practice clinical psychologists in the community), which precluded us undertaking analyses according to their primary site of cancer care. Given that we recruited AYA survivors through survivorship clinics, who were on average only a few years post-treatment, our sample does not represent cancer survivors who are lost to follow-up, and further into long-term survivorship, who may have different patterns of HCU. It is likely that being closer to the completion of cancer treatment may afford survivors relatively easier access to services based at, or linked with, their treating hospital. We also did not explore barriers AYAs can experience to accessing care [12], whether care was accessed or not. This limits the conclusions we can draw about the access afforded to survivors through the hospital setting. We also did not collect data on whether or not survivors were experiencing any ongoing late effects; this limits our ability to gauge the appropriateness of reported HCU relative to their needs, and for our survivor group means that we are unable to determine whether their HCU might have been for screening, intervention for late effects/cancer-related sequelae, or for something entirely independent. It is also possible that AYAs who chose to participate in our study were more highly functioning relative to non-respondents. It is also possible that participating AYAs were more motivated to participate in this study. This may reflect that they actually had more difficulties or increased HCU relative to non-respondents. However, data from other studies using similar cohort methodology and cross-sectional designs have shown that young survivors who participated in those studies were representative of the broader survivor population, which provides some confidence in these data [68,69].

Our cross-sectional design means that we cannot draw conclusions about whether HCU had any causal impact on AYAs mental health or general functioning. Rather than healthcare, it is also plausible that we recruited a particularly high-functioning survivor sample, as other studies have noted poorer perceived health among long-term AYA cancer survivors [47]. Alternatively, it could also be that our comparison group, many of whom were tertiary and University students, was experiencing higher rates of untreated psychological distress and stress [1]. Finally, we did not measure a number of individual factors (e.g., health literacy, perceptions of healthcare need, motivation/engagement with healthcare) or sociodemographic factors (e.g., family/social resources and support, financial resources, transportation, living situation and mobility) which may have an important bearing on AYAs’ ability to successfully access and engage with healthcare services [70]. 

### 4.2. Future Directions

#### 4.2.1. Mental Health Support into Long-Term Survivorship

Cancer aside, AYAs aged 18–25 years are the group most likely to experience mental health disorders, yet they are the group for whom this is the least likely to be detected or appropriately treated [71,72]. Our AYA cancer survivors reported relatively low rates of psychological distress. While patterns of distress did not markedly differ further into longer-term cancer survivorship, rates of accessing mental healthcare services did appear to lessen among survivors with greater time since treatment. Many Youth Cancer Services in Australia continue to offer free, hospital-delivered psychological support several years into survivorship, including using telehealth technologies to minimize barriers to accessing this support [58,73]. However, Australian Youth Cancer Services do not all continue to directly offer mental health and psychological support up to and beyond five years post-diagnosis [30], and access to a dedicated psychologist, let alone psychiatry, also remains a challenge for most of the Australian long-term follow-up survivorship care clinics for AYA survivors managed in the pediatric system [27]. As survivors move further from hospital-delivered care, longer-term cancer survivors may therefore experience a similar gap in mental healthcare to their community-based counterparts. Unlike hospital-delivered psychosocial care, which is largely free for cancer survivors, financial cost becomes a significant barrier for AYAs accessing mental health support delivered in the community, for example through private-practice clinical psychologists [74]. 

AYA survivors whose mental healthcare needs remain unmet are a vulnerable subgroup who remain at high risk of poor quality of life [54]. Recent Australian reports have highlighted that when seeking help with mental health issues, AYAs in the general community report greatest preference for consulting with a GP they know and trust for initial support, over other healthcare professionals (such as a school counsellor, telephone counselling line, or adolescent mental health service) [75]. This is also consistent with established frameworks highlighting the core role of primary care in facilitating access to mental healthcare in the community [76]. Our findings indicated that only a minority of AYA survivors (and comparison AYAs) reported having recently engaged with a GP. For survivors, it is possible that further into survivorship, as they move away from relying on hospital-delivered healthcare services, these relatively low rates of GP engagement could become more problematic in terms of facilitating their access to ongoing, community-delivered mental healthcare. It may be appropriate to expect a higher rate of engagement with GPs among survivors relative to their peers without a cancer history, given survivors’ likelihood of late effects emerging even several decades after cancer treatment completion [29]. In fact, recent models of optimal long-term survivorship care for AYAs have advocated for GPs in primary care playing a more active role in the ongoing surveillance and management of survivors’ late effects as they move further away from the hospital system [27,29,77]. Determining optimal pathways to ensure AYA cancer survivors continue to be able to access evidence-based mental healthcare is critical. Given that our study examined a cohort of AYAs prior to the onset of COVID-19, it will be important to examine how exposure to models of remotely-delivered virtual care may change and even enhance how survivors continue to access tailored survivorship care into the future [58,78].

Routine screening and mental health follow-up into long-term survivorship is also critical to continue to address survivors’ psychotropic medication use and needs [73,79]. Our survivors reported less distress yet relatively high rates of psychotropic medication use relative to the community comparison group. The issue of psychotropic medication prescription—and its ongoing monitoring/surveillance—in cancer survivorship highlights the broader problem of whether and how best to transition (mental) healthcare services in survivorship from the acute, specialist hospital setting to community-delivered, general practice settings. De-prescribing, a process of monitoring medication use after prescription and discontinuing where necessary, is particularly salient for AYAs whose mental health needs are expected to greatly evolve over time [80]. Even in adult oncology there is a lack of data on how psychotropic medications are prescribed and monitored among patients over time [63,81]. For example, if a young survivor is prescribed an anti-depressant medication by their oncology team during active cancer treatment, it is not clear whether, when, how, and to whom the responsibility for monitoring this medication use might be transferred. Future studies should explore the appropriateness of medication usage for AYAs in survivorship.

#### 4.2.2. Access versus Accessibility 

Finally, our data must be considered in the context of a broader discussion around how individuals successfully access healthcare services. Based on a wealth of data documenting AYAs’ unique developmental and healthcare-related needs, recent international literature has emphasized the development of youth-friendly, age-appropriate cancer services for AYAs [19,20,23,25,30,82]. Recent data have highlighted that AYA-specific, age-appropriate health services are a common (unmet) need [20,34,83], and that having access to such services and fewer unmet health- and healthcare-related needs may lead to less distress and better quality of life [21,24,54]. The sample of AYAs in this study was linked to a large metropolitan hospital site with a specialist AYA service, which may have meant that our survivors had better access to age-appropriate health services relative to other Australian survivors. This may reflect the high-quality of a tailored, age-appropriate, AYA-focused model of cancer care. When stratified by survivors’ ongoing level of medical risks into longer-term survivorship, such models may help bridge the gap between what survivors want, need, and what survivorship care is able to provide them with [27,32].

There is a lack of evidence-based strategies with demonstrated effectiveness in enhancing AYAs’ access to primary and community-delivered healthcare [84,85]. Ultimately, even optimally-designed, youth-friendly healthcare services are likely not enough on their own to bridge all of the barriers to accessing healthcare that are likely exist for AYA cancer survivors. Successful healthcare access relies on an interaction between the characteristics of healthcare services themselves, and characteristics of the individuals trying to access them, including their ability to perceive their healthcare needs, seek and reach the healthcare services, pay for the healthcare services, and ultimately engage with them [70]. As a group, AYAs may be at risk for poor health literacy [14,15], and prior studies have shown links between lower education and suboptimal HCU in survivorship, even when survivors are experiencing severe or life-threatening late effects [51,52]. AYAs must also *want* to engage with healthcare services: recent Australian reports indicate that AYAs are most likely to prefer to turn to friends and family for help with mental health concerns, over more formal avenues of support [86]. Family resources and supports—which may include family-level health literacy [87] and the provision of logistical supports by parents/caregivers such as transport to appointments and financial support [88]—are also likely to be critical to facilitating survivors’ ongoing engagement with healthcare. Future research is needed to examine how AYA and family resources, levels of health literacy, and levels of trust and engagement with the healthcare system, may impact the extent to which AYAs proactively seek out, and subsequently use these services. Whether accessing such AYA-targeted services ultimately leads to a better ‘match’ between recommended and appropriate HCU offered to—and actually taken up by—survivors is a topic for future research.

## 5. Conclusions

Our survivor cohort reported higher HCU across multiple domains compared with a community-based comparison group. Relative to the comparison group, survivors reported less distress in terms of depression and anxiety symptoms, and showed positive general functioning, including similar work/study participation, similar perceived health-related quality of life, and greater engagement with hobbies and social activities. Several survivor subgroups, including females, those with brain/solid tumors, and those who had finished treatment more recently, reported greater recent use of healthcare. Greater HCU was linked with better perceived health-related quality of life. Understanding how to engage AYA survivors in survivorship care that is appropriate, tailored to their needs, and delivered in a way they are motivated and able to access remains a challenge for the field. 

## Figures and Tables

**Table 1:** Battery of self-reported measures used.

Domain	Measure and Subscale Information	Scoring and Analysis Information	Psychometric Validity Data Available
Demographic characteristics	Adolescents and young adults (AYAs) age, sex, level of educational attainment, employment status, family structure, cancer diagnosis, treatment regimen	We dichotomized reported educational attainment (achievement below or at/above Year 12, the final year of high school in Australia) and cancer diagnosis (grouping blood cancers [i.e., leukemias, lymphomas] vs. all other cancer types [i.e., brain/solid tumors].	-
Mental health	Depression, Anxiety, Stress Scales-21 item short form (DASS-21): depression (7 item) and anxiety (7 item) subscales	4-point scale, rating extent to which they had experienced each symptom in the past week (0 = “Did not apply to me at all—NEVER” to 3 = “Applied to me very much, or most of the time—ALMOST ALWAYS”). Higher responses indicate more severe symptoms.	Used in Australian adolescents [39], cancer patients [40] and AYAs [39,41], with strong internal consistency and reliability [39,41].
Health-related quality of life	Short Form-Six Dimension (SF6D): a six-dimensional health status classification derived from the 36-Item Short Form Health Survey (SF-36) questionnaire [42]	Measures self-reported overall perceived health status, on a 5-item scale (“In general, would you say your health is: …poor, fair, good, very good, excellent”). For the purposes of analysis, we dichotomized participants’ responses into a binary outcome (fair-poor, good-excellent).	The use of this single item is a common approach to minimize participant burden and is considered valid, sensitive and reliable [43]
General functioning	Time taken off from study/work. Reasons included “Sickness or feeling unwell”, “Tiredness or low energy”,“Low motivation or ‘feeling flat’”, “Medical or health-related appointments”, “Unable to keep up with the workload”, “Self-conscious about physical scars or changes”, “Difficulty getting on with friends/colleagues”, and “Other”	Estimated days absent over the past 4 weeks, and the main reasons for this. Where participants selected “Other” they were asked to specify this in free-text.	-
	Engagement with productive activities: including ‘Paid work of any kind’, ‘Study or learning of any kind (school, university, TAFE, other courses)’, ‘Exercise or sports’, ‘Personal hobbies (e.g., art, music, films, books, outdoor activities, cooking)’, ‘Socializing with friends’, and ‘Socializing with other young people [with cancer] (includes connecting online)’ (study-developed)	Estimated days engaged in any of these productive activities over the past 4 weeks. Engagement in productive activities: the item “Socialising with other young people with cancer (includes connecting online)” was reworded so as not to refer to cancer for control participants.	-
Self-reported healthcare use (HCU) for survivorship support	Medical HCU: included seeing a general practitioner, oncologist/radiation oncologist, nurse in hospital, nurse in community, or fertility specialist. Hospital-delivered HCU: emergency department visits or hospital admissions. Mental HCU: included psychologists, social workers, counselors, psychiatrists, and community-delivered cancer support and/or mental health support organizations	Health professionals/services accessed for support over the past six months. For the purposes of our analysis, participants’ health services use was assessed according to frequency of use (not cost) by profession, as well as across total, general, and mental health service use categories.	-
	Medication use: Any medications/supplements taken, and the reasons for their use, over the past six months. Participants were not limited to listing only prescribed medications but were encouraged to omit very occasional medication use (e.g., an occasional dose of paracetamol)	Free-text response. Use was reported according to the number and classification of medications (not cost). The classification of these medications was manually checked by a senior pediatric oncologist (author RJC), with reference to the Monthly Index of Medical Specialties online database.	-

**Table 2:** Participant sociodemographic characteristics across groups.

		Cancer Survivors (*n* = 93)	Comparison (*n* = 183)	Total (*n* = 276)	*p*-Value
Age (range: 15–31 years)—M (SD)		21.9 (3.53)	19.7 (3.16)	20.5 (3.44)	**<0.001**
**Sex**	Female	52 (55.9%)	129 (70.5%)	181 (65.6%)	**0.036**
Highest education level attained	Year 12/below ^1^	45 (48.4%)	160 (87.4%)	205 (74.3%)	**<0.001**
	Above Year 12 ^2^	48 (51.6%)	23 (12.6%)	71 (25.7%)	
Currently in employment, education and/or training	Yes	81 (87.1%)	173 (94.5%)	254 (92.0%)	**0.031**
Socioeconomic status (Index of Relative Socioeconomic Disadvantage Decile)	1	6 (6.5%)	14 (7.9%)	20 (7.4%)	**0.028**
	2	5 (5.4%)	7 (4.0%)	12 (4.4%)	
	3	5 (5.4%)	1 (0.6%)	6 (2.2%)	
	4	8 (8.6%)	11 (6.2%)	19 (7.0%)	
	5	11 (11.8%)	5 (2.8%)	16 (5.9%)	
	6	9 (9.7%)	18 (10.2%)	27 (10.0%)	
	7	8 (8.6%)	16 (9.0%)	24 (8.9%)	
	8	9 (9.7%)	19 (10.7%)	28 (10.4%)	
	9	12 (12.9%)	36 (20.3%)	48 (17.8%)	
	10	20 (21.5%)	50 (28.2%)	70 (25.9%)	
Parents separated/divorced	Yes	30 (32.3%)	36 (19.7%)	66 (23.9%)	**0.024**
Has siblings	Yes	82 (88.2%)	161 (88.0%)	243 (88.0%)	0.963
Born in Australia ^	Yes	84 (90.3%)	128 (69.9%)	212 (77.1)	**<0.001**
Aboriginal and/or Torres Strait Islander descent	Yes	1 (1.1%)	3 (1.6%)	4 (0.7%)	0.534
LOTE at home *^^*	Yes ^	13 (14.0%)	93 (50.8%)	106 (38.4%)	**<0.001**
**Cancer-related characteristics (survivor group only; *n* = 93)**					
Age at cancer diagnosis (years)		M = 14.7 (SD = 6.59); Median = 16.0; IQR = 8.5, Range: 0–27	N/A	N/A	-
Time since diagnosis (years)		Median = 3.0; IQR = 11.0, Range:0–26.6	N/A	N/A	-
Cancer diagnosis category	Blood	51 (54.8%)	N/A	N/A	-
	Solid tumor	29 (31.2%)	N/A	N/A	-
	Brain	11 (11.8%)	N/A	N/A	-
	Not sure	2 (2.2%)	N/A	N/A	-
Treatments received	Surgical	51 (54.8%)	N/A	N/A	-
	Chemotherapy	83 (89.2%)	N/A	N/A	-
	Radiotherapy	43 (46.2%)	N/A	N/A	-
	Bone marrow/stem cell transplant	19 (20.4%)	N/A	N/A	-
Relapse (ever recurred/relapsed)		12 (12.9%)	N/A	N/A	-
Time since treatment (months)		Median = 21.5; IQR = 119.25 Range: 1–309	N/A	N/A	-

Bold *p*-values denote statistical significance at the level of *p* < 0.05. Abbreviations: AYA = adolescent and young adult; M = mean; N/A = not applicable; SD = standard deviation; IQR = interquartile range; LOTE = Language other than English. ^1^ Within the Australian education system, Year 12 is the final year of high school prior to tertiary education. ^2^ ‘Year 12 and above’ included all AYAs who had at least completed their higher-school certificate, as well as AYAs who had completed further studies beyond that including undergraduate, postgraduate university studies, and Technical And Further Education (TAFE) qualifications. Higher decile = more socioeconomic disadvantage ^. Overall, the three most common countries of birth other than Australia were China (*n* = 10, 3.6%), New Zealand (*n* = 6, 2.2%), and England, South Africa, Singapore, Hong Kong, and Indonesia (*n* = 4, 1.4%). Additionally, of our control group, *n* = 23 reported that they were not Australian/New Zealand citizens. Of these, *n* = 4 (2.2%) reported that they were staying in Australia on a permanent residency visa, and *n* = 19 (10.4%) reported that they were on student visas. This question was not asked of our cancer group. ^^ The three most common languages spoken at home were Mandarin (*n* = 21, 7.7%), Cantonese (*n* = 17, 6.2%), and Vietnamese (*n* = 10, 3.6%). Cancer categorizations. Blood cancers: Acute Myeloid Leukemia, Acute Lymphoblastic Leukemia, Fanconi’s anemia, Hodgkin’s Lymphoma, and Non-Hodgkin’s Lymphoma; Solid tumors included bone and soft tissue sarcomas, liposarcoma, seminoma, Wilms’ tumor, hepatoblastoma, breast cancer, ovarian cancer, yolk sac tumor, testicular cancer, submandibular mammary carcinoma, fibrolamella hepatocellular carcinoma, clear cell cervical cancer, metastatic neuroendocrine carcinoid tumor of the appendix; neuroblastomas. Brain cancers: medulloblastomas and other brain/central nervous system cancers.

**Table 3:** Comparison of overall, medical and mental healthcare use as well as medication use reported between groups over the past six months (*n* = 276).

		Cancer Survivors (*n* = 93)	Comparison (*n* = 183)	*χ^2^*	*p*-Value
Overall HCU	Yes	61 (65.6%)	110 (60.1%)	0.786	0.375
Hospital-delivered HCU ^1^	Total	29 (31.2%)	37 (20.3%)	4.074	**0.044**
	ED admission	23 (24.7%)	24 (13.1%)	5.889	**0.015**
	Hospital admission	15 (16.1%)	17 (9.3%)	2.814	0.093
	Oncologist/Radiation oncologist ^2^	19 (20.7%)	N/A	N/A	N/A
Medical HCU ^3^	Total	61 (65.6%)	86 (47.0%)	8.567	**0.003**
	GP	32 (34.8%)	65 (35.5%)	0.015	0.904
	Nurse in hospital/community	12 (13.0%)	N/A	N/A	N/A
	Fertility specialist	12 (13.0%)	N/A	N/A	N/A
	Other health professionals	11 (12.0%)	14 (7.7%)	1.374	0.241
Mental HCU	Total	49 (52.7%)	43 (23.5%)	23.645	**<0.001**
	Psychologist	33 (35.5%)	30 (16.4%)	12.757	**<0.001**
	Social worker	32 (34.4%)	14 (7.7%)	31.789	**<0.001**
	Counsellor	18 (19.4%)	26 (14.2%)	1.219	0.270
	Psychiatrist	10 (10.8%)	14 (7.7%)	0.748	0.387
	Community mental health/cancer support organization	25 (27.2%)	24 (13.1%)	8.264	**0.004**
Reported ≥ 1 medication used		57 (61.3%)	77 (42.1%)	9.113	0.003
Average number of medications used, M (SD)		1.7 (2.35)	0.7 (1.08)	T = 10.622	<0.001

Bold *p*-values denote statistical significance at the level of *p* < 0.05. ^1^ Total hospital-delivered HCU included emergency department, hospital admissions, and oncologist/radiation oncologist service use; ^2^ total medical HCU included all hospital based HCU as well as GP, nurse, fertility specialist, and ‘other’ health professionals. ^3^ Several professionals including oncologists were not included in the comparison group survey. Abbreviations: ED = Emergency Department; GP = general practitioner; HCU = healthcare use; M = mean; SD = standard deviation.

**Table 4:** Patterns of healthcare use reported by cancer survivors, according to age at diagnosis and time since treatment (*n* = 93).

		Age at Diagnosis ^a^				Number of Years Post-Treatment ^b^				
		Pediatric (*n* = 35)	AYA (*n* = 58)	*χ^2^*	*p*-Value	1 (*n* = 42)	2–4 (*n* = 22)	5^+^ (*n* = 29)	*χ^2^*	*p*-Value
Overall HCU	Yes	12 (34.3%)	49 (84.5%)	24.370	**<0.001**	38 (90.5%)	15 (68.2%)	8 (27.6%)	30.770	**<0.001**
Hospital-delivered HCU ^1^	Total	8 (22.9%)	21 (36.2%)	1.813	0.178	16 (38.1%)	7 (31.8%)	6 (20.7%)	6.427	0.093
	ED admission	4 (11.4%)	19 (32.8%)	5.335	**0.021**	14 (33.3%)	6 (27.3%)	3 (10.3%)	7.383	0.061
	Hospital admission	5 (14.3%)	10 (17.2%)	0.141	0.707	10 (23.8%)	1 (4.5%)	4 (13.8%)	4.616	0.202
	Oncologist/Radiation oncologist ^3^	8 (22.9%)	22 (37.9%)	2.445	0.118	19 (45.2%)	7 (31.8%)	4 (13.8%)	9.302	**0.026**
Medical HCU ^2^	Total	17 (48.6%)	44 (75.9%)	24.370	**<0.001**	33 (78.6%)	15 (68.2%)	13 (44.8%)	9.362	**0.025**
	GP	13 (37.1%)	19 (32.8%)	0.139	0.710	14 (33.3%)	9 (40.9%)	9 (31.0%)	0.635	0.888
	Nurse in hospital/community	2 (5.7%)	17 (29.3%)	7.692	**0.012**	17 (40.5%)	1 (4.5%)	1 (3.4%)	19.749	**<0.001**
	Fertility specialist	1 (2.9%)	11 (19.0%)	5.168	**0.023**	8 (19.0%)	2 (9.0%)	2 (6.9%)	3.892	0.273
	Other health professionals	1 (2.9%)	10 (17.2%)	4.443	**0.035**	9 (21.4%)	1 (4.5%)	1 (3.4%)	7.332	0.062
Mental HCU	Total	10 (28.6%)	39 (67.2%)	13.094	**<0.001**	31 (73.8%)	10 (45.5%)	8 (27.6%)	18.058	**<0.001**
	Psychologist	7 (20.0%)	26 (44.8%)	5.877	**0.015**	22 (52.4%)	6 (27.3%)	5 (17.2%)	10.346	**0.016**
	Social worker	4 (11.4%)	28 (48.3%)	13.131	**<0.001**	25 (59.5%)	4 (18.2%)	3 (10.3%)	26.400	**<0.001**
	Counsellor	2 (5.7%)	16 (27.6%)	6.690	**0.010**	13 (35.7%)	1 (4.5%)	2 (6.9%)	13.597	**0.004**
	Psychiatrist	2 (5.7%)	3 (13.8%)	1.485	0.223	6 (14.3%)	1 (4.5%)	3 (10.3%)	2.119	0.548
	Community mental health/cancer support organization	3 (8.6%)	22 (38.6%)	9.878	**0.002**	19 (45.2%)	5 (22.7%)	1 (3.4%)	16.946	**0.001**
Reported ≥ 1 medication used		21 (60.0%)	36 (62.1%)	0.039	0.843	25 (59.5%)	15 (68.2%)	17(58.6%)	1.174	0.759
Average number of medications used—M (SD)		1.8 (2.29)	1.8 (2.41)	t = −0.303	0.763	1.8 (2.29)	1.8 (2.68)	1.7 (2.26)		

Bold *p*-values denote statistical significance at the level of *p* < 0.05. ^a^ Age at diagnosis: pediatric = diagnosed <15 years; AYA= 15 years and above. ^b^ Years post-treatment completion: 1st year, 0–12 months ago, inclusive; 2nd year–4th years post-treatment: 13–59 months inclusive; 5th year post-treatment: ≥60 months post-treatment. ^1^ Total hospital-delivered HCU included emergency department, hospital admissions, and oncologist/radiation oncologist service use; ^2^ total medical HCU included all hospital based HCU as well as GP, nurse, fertility specialist, and ‘other’ health professionals. ^3^ Several professionals including oncologists were not included in the comparison group survey. Abbreviations: AYA = adolescent and young adult; ED = Emergency Department; GP = general practitioner; HCU = healthcare use; M = mean; SD = standard deviation. t= t-test statistic.

**Table 5:** Summary of univariable and multivariable regressions predicting likelihood of total healthcare use (HCU), and in medical, mental health, and medication domains among cancer survivors (*n* = 93).

		Univariable Regression			Multivariable Regression			
		OR	95%CI	*p*-Value	OR	95%CI	*p*-Value	R Square
Total HCU								
Sociodemographic predictors	Sex (female vs. male)	0.99	0.41–2.32	0.962	1.57	0.34–7.23	0.563	0.503 ^a^/0.696 ^b^
	Employment status (working/studying vs. not)	0.15	0.02–1.19	0.073	0.27	0.02–3.53	0.315	
	Speaks a language other than English at home	1.21	0.34–4.29	0.766	2.47	0.36–17.05	0.358	
Cancer-related predictors	Age at diagnosis (in years)	1.21	1.11–1.32	**<0.001**	0.72	0.55–0.96	**0.023**	
	Diagnosis (non-blood vs. blood cancers)	1.16	0.49–2.76	0.737	9.79	1.48–64.87	**0.018**	
	Time since treatment (per month)	0.98	0.97–0.99	**<0.001**	0.95	0.92–0.98	**<0.001**	
Medical HCU								
Sociodemographic predictors	Sex (female vs. male)	2.12	0.89–5.07	0.089	4.38	1.42–13.58	**0.010**	0.219 ^a^/0.305 ^b^
	Employment status (working/studying vs. not)	0.15	0.02–1.19	0.073	0.11	0.01–1.19	0.069	
	Speaks a language other than English at home	1.21	0.34–4.29	0.766	2.61	0.51–13.36	0.250	
Cancer-related predictors	Age at diagnosis (in years)	1.11	1.04–1.19	**0.003**	1.10	0.91–1.32	0.321	
	Diagnosis (non-blood vs. blood cancers)	0.96	0.40–2.27	0.917	1.72	0.57–5.16	0.333	
	Time since treatment (per month)	0.99	0.99–0.99	**0.003**	1.00	0.99–1.01	**0.825**	
Mental HCU								
Sociodemographic predictors	Sex (female vs. male)	0.93	0.41–2.12	0.868	0.97	0.36–2.61	0.958	0.186 ^a^/0.248 ^b^
	Employment status (working/studying vs. not)	0.77	0.23–2.63	0.675	0.93	0.21–4.06	0.920	
	Speaks a language other than English at home	1.06	−0.33–3.42	0.928	1.03	0.26–4.11	0.970	
Cancer-related predictors	Age at diagnosis (in years)	1.12	1.04–1.20	**0.002**	0.95	0.81–1.12	0.573	
	Diagnosis (non-blood vs. blood cancers)	1.68	0.73–3.86	0.221	3.24	1.12–9.35	**0.030**	
	Time since treatment (per month)	0.99	0.99–1.00	0.002	0.99	0.97–1.00	0.056	
Medication use ^1^								
Sociodemographic predictors	Sex (female vs. male)	0.49	0.21–1.12	0.092	0.57	0.24–1.39	0.219	0.046 ^a^/0.062 ^b^
	Employment status (working/studying vs. not)	1.19	0.35–4.00	0.780	0.77	0.20–2.97	0.703	
	Speaks a language other than English at home	0.49	0.15–1.62	0.239	0.70	0.19–2.58	0.596	
Cancer-related predictors	Age at diagnosis (in years)	1.02	0.96–1.08	0.576	1.07	0.94–1.22	0.321	
	Diagnosis (non-blood vs. blood cancers)	1.09	0.48–2.49	0.832	1.31	0.53–3.20	0.561	
	Time since treatment (per month)	1.00	1.00–1.01	0.726	1.00	0.99–1.01	0.358	

Bold *p*-values denote statistical significance at the level of *p* < 0.05. ^1^ Medication use excluding medication-based contraceptives, as this was only reported by females. ^a^ Cox and Snell R^2^; ^b^ Nagelkerke R^2^ Abbreviations: CI = confidence interval; OR = odds ratio.

**Table 6:** Multivariable Poisson regressions predicting the total number of types of HCU cancer survivors accessed within each category.

		RR	95%CI RR	Wald χ^2^	*p*
Total HCU				29.80	0.000
Sociodemographic predictors	Sex (female vs. male)	1.06	0.71, 1.58	0.09	0.763
	Employment status (working/studying vs. not)	0.76	0.45, 1.28	1.06	0.304
	English spoken at home vs. not	1.49	0.89, 2.49	2.33	0.127
Cancer-related predictors	Age at diagnosis (in years)	0.99	0.93, 1.05	0.20	0.651
	Diagnosis (non-blood vs. blood cancers)	1.27	0.86, 1.87	1.43	0.232
	Time since treatment (per month)	0.99	0.99, 1.00	9.71	** 0.002 **
Hospital-delivered HCU				23.41	**0.001**
Sociodemographic predictors	Sex (female vs. male)	1.58	0.98, 2.55	3.53	0.060
	Employment status (working/studying vs. not)	0.77	0.43, 1.39	0.74	0.389
	English spoken at home vs. not	0.93	0.46, 1.86	0.05	0.832
Cancer-related predictors	Age at diagnosis (in years)	0.98	0.92, 1.05	0.29	0.590
	Diagnosis (non-blood vs. blood cancers)	1.21	0.78, 1.90	0.71	0.401
	Time since treatment (per month)	0.99	0.98, 0.99	7.94	** 0.005 **
Medical HCU				23.76	**0.001**
Sociodemographic predictors	Sex (female vs. male)	1.47	0.97, 2.23	3.29	0.070
	Employment status (working/studying vs. not)	0.65	0.39, 1.08	2.74	0.098
	English spoken at home vs. not	1.36	0.79, 2.36	1.23	0.268
Cancer-related predictors	Age at diagnosis (in years)	0.98	0.93, 1.04	0.32	0.570
	Diagnosis (non-blood vs. blood cancers)	1.06	0.71, 1.59	0.09	0.770
	Time since treatment (per month)	0.99	0.98, 0.99	7.19	** 0.007 **
Mental HCU				20.09	**0.003**
Sociodemographic predictors	Sex (female vs. male)	1.11	0.70,1.75	0.20	0.652
	Employment status (working/studying vs. not)	0.88	0.47, 1.65	0.15	0.697
	English spoken at home vs. not	1.26	0.69, 2.32	0.57	0.450
Cancer-related predictors	Age at diagnosis (in years)	0.98	0.92, 1.05	0.27	0.607
	Diagnosis (non-blood vs. blood cancers)	1.38	0.89, 2.16	2.06	0.151
	Time since treatment (per month)	0.99	0.98, 0.99	7.41	** 0.006 **
Medication use				3.06	0.802
Sociodemographic predictors	Sex (female vs. male)	1.40	0.73, 2.68	1.01	0.316
	Employment status (working/studying vs. not)	1.07	0.40, 2.82	0.02	0.899
	English spoken at home vs. not	0.76	0.27, 2.17	0.26	0.613
Cancer-related predictors	Age at diagnosis (in years)	1.05	0.96, 1.14	1.02	0.311
	Diagnosis (non-blood vs. blood cancers)	1.01	0.54, 1.88	0.00	0.981
	Time since treatment (per month)	1.00	1.00, 1.01	0.43	0.514

Bold *p*-values denote statistical significance at the level of *p* < 0.05. Abbreviations: CI = confidence interval; HCU = healthcare use; RR = relative rate of use.

**Table 7:** Mental health, perceived health-related quality of life, and general functioning outcomes across groups.

		Cancer Survivors (*n* = 93)	Comparison (*n* = 183)	Total (*n* = 276)	*p*-Value
Health-related quality of life	Good/very good/excellent	37 (39.8%)	86 (47.0%)	123 (44.6%)	0.255
	Poor/fair	56 (60.2%)	97 (53.0%)	153 (55.4%)	
Overall anxiety level ^1^ (DASS-21)	Normal	79 (84.9%)	139 (76.0%)	218 (79.0%)	
	Mild	10 (10.8%)	23 (12.6%)	33 (12.0%)	
	Moderate	4 (4.3%)	15 (8.2%)	19 (6.9%)	
	Severe	0 (0.0%)	6 (3.3%)	6 (2.2%)	
	Total scores M (SD)	4.8 (5.7)	7.9 (7.5)	6.8 (7.1)	**0.001**
Overall depression level ^1^ (DASS-21)	Normal	75 (80.6%)	130 (71.4%)	205 (74.5%)	
	Mild	9 (9.7%)	29 (15.9%)	38 (13.8%)	
	Moderate	7 (7.5%)	19 (10.4%)	26 (9.5%)	
	Severe	2 (2.2%)	2 (1.1%)	4 (1.5%)	
	Extremely severe	0 (0.0%)	2 (1.1%)	2 (0.7%)	
	Total scores M (SD)	5.9 (7.5)	9.0 (8.0)	7.9 (8.0)	**0.003**
Overall missed study/work	Took days off work/study—N (%)	45 (47.3%)	92 (54.1%)	137 (49.6%)	0.767
Total days absent	M (SD)	3.1 (6.6)	2.2 (4.1)	2.5 (5.1)	0.910
Reasons for missed study/work ^2^	Sickness or feeling unwell	25 (56.8%)	43 (43.4%)	68 (47.2%)	0.117
	Tiredness or low energy	8 (18.2%)	37 (37.4%)	45 (31.5%)	**0.023**
	Low motivation or ‘feeling flat’	3 (6.8%)	19 (19.2%)	22 (15.4%)	0.058
	Medical or health-related appointments	2 (4.5%)	4 (4.0%)	6 (4.2%)	0.889
	Unable to keep up with the workload	16 (36.4%)	54 (54.5%)	70 (49.0%)	**0.045**
	Self-conscious about physical scars or changes	18 (40.9%)	9 (9.1%)	27 (18.9%)	**<0.001**
	Difficulty in getting on with friends/colleagues	2 (4.5%)	10 (10.1%)	12 (8.4%)	0.269
	Others	5 (11.4%)	11 (11.1%)	16 (11.2%)	0.965
Days engaged in activities	M (SD)	15.0 (6.3)	12.4 (5.6)	13.3 (5.9)	**0.001**
Number of AYAs engaged in different activities N (%)	Paid work	55 (59.1%)	110 (60.1%)	165 (59.8%)	0.897
	Study	46 (49.5%)	162 (88.5%)	208 (75.4%)	**<0.001**
	Exercise or sports	79 (84.9%)	134 (73.2%)	213 (77.2%)	**0.028**
	Personal hobbies	84 (90.3%)	137 (74.9%)	221 (80.1%)	**0.002**
	Socializing with friends ^3^	85 (91.4%)	156 (85.2%)	241 (87.3%)	0.147
	Socializing with peers with cancer ^4^	20 (21.5%)	N/A	N/A	-
Days of engagement: by activity	Paid work	14.6 (7.4)	9.9 (6.4)	11.5 (7.1)	**<0.001**
	Study	13.6 (7.9)	11.3 (6.6)	11.8 (7.0)	0.094
	Exercise or sports	11.4 (8.1)	10.4 (7.3)	10.7 (7.6)	0.445
	Personal hobbies	17.1 (9.8)	14.1 (8.9)	15.2 (9.3)	**0.027**
	Socializing with friends ^2^	19.1 (9.5)	17.2 (9.6)	17.8 (9.6)	0.130
	Socializing with cancer peers ^3^	5.8 (7.0)	N/A	N/A	-
Participated in group social activities	N (%)	42 (45.2%)	114 (62.3%)	156 (56.5%)	**0.007**

Bold *p*-values denote statistical significance at the level of *p* < 0.05. ^1^ The majority (8/10) of cancer survivor AYAs with higher-range (moderate-extremely severe) DASS-21 scores were within the first five years post-diagnosis. ^2^ Reasons for missed study/work calculated based on the denominator of AYAs who indicated that they had taken any days off work/study. ^3^ Socializing included connecting online, e.g., via social media. ^4^ AYAs with a cancer history were also asked about socializing specifically with other young people with cancer (including connecting online). Abbreviations: AYA = adolescent and young adult; M = mean; SD = standard deviation; DASS-21 = Depression, Anxiety, Stress Scales-Short Form.

**Table 8:** Types of medication used in the past six months, by group.

	Cancer Survivors (*n* = 93)		Comparison (*n* = 183)		Total (*n* = 276)	
Any medication ^1^	57	61.3%	77	42.1%	134	48.6%
Vitamins and supplements	17	18.3%	31	16.9%	48	17.4%
Anti-depressants/anxiety	11	11.8%	10	5.5%	21	7.6%
Contraceptive ^2^	10	19.2%	23	16.9%	33	18.1%
Hormone therapy	9	9.7%	4	2.2%	13	4.7%
Pain killer	7	7.5%	2	1.1%	9	3.3%
Cancer treatment-related medications ^3^	5	5.4%	0	0.0%	5	1.8%
Asthma	4	4.3%	3	1.6%	7	2.5%
Steroids	4	4.3%	0	0.0%	4	1.4%
Antibiotics ^4^	4	4.3%	7	3.8%	11	4.0%
Anti-reflux	3	3.2%	2	1.1%	5	1.8%
Insomnia	3	3.2%	8	4.4%	11	4.0%
ADHD	2	2.2%	4	2.2%	6	2.2%
Anti-seizure	2	2.2%	0	0.0%	2	0.7%
Anti-fungal	2	2.2%	0	0.0%	2	0.7%
Dyslipidemic agents	2	2.2%	0	0.0%	2	0.7%
Antihypertension	1	1.1%	2	1.1%	3	1.1%
Constipation	1	1.1%	0	0.0%	1	0.4%
Anti-histamine	1	1.1%	3	1.6%	4	1.4%

Note. ^1^ Number reporting at least one medication of any kind. ^2^ Medication-based contraceptive use was calculated as a proportion of female participants within each group as no male respondents reported this, which was *n* = 52 female cancer survivors and *n* = 130 female comparison AYAs, respectively. ^3^ Due to the broad period of survivorship our study captured, a small number of our survivors (5.4%) continued to take medications related to their cancer treatment, including related to leukemia maintenance treatment (e.g., methotrexate), or prophylactic treatment for graft-versus-host disease (e.g., cyclosporine). ^4^ Antibiotics are commonly prescribed for survivors across the first six months following active treatment completion. Abbreviations: ADHD = Attention Deficit and Hyperactivity Disorder.

## Data Availability

The data presented in this study are available upon reasonable request from the corresponding author, as is the full study protocol and the study materials. The data are not publicly available due to restrictions within the ethical approval.

## Supplementary Materials

The following materials are available online at www.mdpi.com/article/10.3390/cancers13215270/s1, Table S1. Proportion of AYAs who reported having accessed mental healthcare services according to participant group and DASS-21 symptom score severity; Table S2. Patterns of psychotropic medication use reported by adolescents and young adults, according to other forms of mental healthcare services they reported accessing; Table S3. Multivariable linear regressions examining relationships between healthcare use (HCU) and general functioning outcomes.
