# Supplementary material for: Patterns and Predictors of Healthcare Use among Adolescent and Young Adult Cancer Survivors versus a Community Comparison Group
Source: Cancers (Basel). 2021 Oct 20;13(21):5270. doi: 10.3390/cancers13215270 (PMC8582416; doi:10.3390/cancers13215270)
Supplement: Supplementary file 1 [file cancers-13-05270-s001.zip › cancers-1352042-supplementary.pdf]

SUPPLEMENTARY MATERIALS

TABLE S1. Proportion of AYAs who reported having accessed mental healthcare services according to participant group and DASS-21 symptom score severity

		Cancer survivors (n=93)		Comparisons (n=183)		Total (N=276)		TOTAL USE
**Service was accessed**	**DASS-21 Scores**	**Low[1] (n=83)**	**High[2] (n=10)**	**Low[1] (n=149)**	**High[2] (n=34)**	**Low[1] (n=232)**	**High[2] (n=44)**	**(N=276)**
**Overall mental HCU**	N *(%)*	41 *(49.4%)*	8 *(80.0%)*	27 *(18.1%)*	16 *(47.1%)*	68 *(29.3%)*	24 *(54.5%)*	92 *(33.3%)*
**Psychologist**	N *(%)*	27 *(32.5%)*	6 *(60.0%)*	22 *(14.8%)*	8 *(23.5%)*	49 *(21.1%)*	14 *(31.8%)*	63 *(22.8%)*
**Psychiatrist**	N *(%)*	8 *(9.6%)*	2 *(20.0%)*	11 *(7.4%)*	3 *(8.8%)*	19 *(8.2%)*	5 *(11.4%)*	24 *(8.7%)*
**Social worker**	N *(%)*	27 *(32.5%)*	5 *(50.0%)*	12 *(8.1%)*	2 *(5.9%)*	39 *(16.8%)*	7 *(15.9%)*	46 *(16.7%)*
**Counsellor**	N *(%)*	16 *(19.3%)*	2 *(20.0%)*	19 *(12.8%)*	7 *(20.6%)*	35 *(15.1%)*	9 *(20.5%)*	44 *(15.9%)*
**GP**	N *(%)*	28 *(34.1%)*	4 *(40.0%)*	50 *(33.6%)*	15 *(44.1%)*	78 *(33.8%)*	19 *(43.2%)*	97 *(35.3%)*
**Psychotropic medications**	N *(%)*	8 *(9.6%)*	3 *(30.0%)*	4 *(2.7%)*	6 *(17.6%)*	12 *(5.2%)*	9 *(20.5%)*	21 *(7.6%)*

DASS-21=Depression, Anxiety, Stress Scales-Short Form. Low[1] = where AYAs scored in the normal-mild range of symptoms for both their DASS-21 depression and anxiety subscale scores; High[2]= where AYAs scored in the moderate, severe or extremely severe ranges for either or both their DASS-21 depression and anxiety subscale scores. HCU=Healthcare Use. GP=General Practitioner

TABLE S2. Patterns of psychotropic medication use reported by adolescents and young adults, according to other forms of mental healthcare services they reported accessing

		Cancer survivors (n=93)		Comparisons (n=183)		Total (N=276)		SIG^
**Service use**	**Psychotropic medication use**	**No (n=82)**	**Yes (n=11)**	**No (n=173)**	**Yes (n=10)**	**No (n=255)**	**Yes (n=21)**	
**Overall mental HCU**	N *(%)*	41 *(50.0%)*	8 *(72.7%)*	35 *(20.2%)*	8 *(80.0%)*	76 *(29.8%)*	16 *(76.2%)*	χ^2=18.787, p<.0001
**Psychologist**	N *(%)*	27 *(32.9%)*	6 *(54.5%)*	23 *(13.3%)*	7 *(70.0%)*	*50* *(19.6%)*	13 *(61.9%)*	χ^2=19.705, p<.0001
**Psychiatrist**	N *(%)*	7 *(8.5%)*	3 *(27.3%)*	10 *(5.8%)*	4 *(40.0%)*	17 *(6.7%)*	7 *(33.3%)*	χ^2=17.378, p<.0001
**GP**	N *(%)*	24 *(29.3%)*	8 *(72.3%)*	55 *(31.8%)*	10 *(100%)*	79 *(31.0%)*	18 *(85.7%)*	χ^2=25.338, p<.0001

SIG=Significance of chi-square test across overall sample.

TABLE S3. Multivariable linear regressions examining relationships between healthcare use (HCU) and general functioning outcomes

		B	SE *B*	*t*	*p*	*F*
**Perceived health-related quality of life[a]**					**.008**	**2.977**
**Sociodemographic predictors**	Sex	.069	.194	0.357	.722	-
	Employment status	.331	.296	1.117	.267	-
	LOTE	.432	.285	1.516	.133	-
**Cancer-related predictors**	Age at diagnosis	.012	.028	0.446	.657	-
	**Diagnosis (Blood cancer vs. other)**	**-.408**	**.196**	**-2.078**	**.041**	**-**
	Time since treatment	.002	.002	0.799	.427	-
**Overall HCU**	**Total number of services used**	**-.084**	**.033**	**-2.558**	**.012**	
**Work and study engagement[b]**					**.001**	**4.066**
**Sociodemographic predictors**	**Sex**	**.169**	**.075**	**2.252**	**.027**	**-**
	LOTE	.000	.108	-0.001	.999	-
**Cancer-related predictors**	Age at diagnosis	.009	.011	0.833	.407	-
	Diagnosis (Blood cancer vs. other)	-.131	.076	-1.725	.088	-
	Time since treatment	.001	.001	1.577	.119	-
**Overall HCU**	**Total number of services used**	**-.029**	**.013**	**-2.256**	**.027**	

Note. a) Perceived health-related quality of life: higher scores reflected poorer perceived health-status; b) Work and study engagement refers to number of self-reported days engaged in either work and/or study during the past four weeks. Age at diagnosis coded in years; Time since treatment completion coded in months.
